# Unraveling Li growth kinetics in solid electrolytes due to electron beam charging

**DOI:** 10.1126/sciadv.abq3285

**Published:** 2023-04-26

**Authors:** Xinxing Peng, Qingsong Tu, Yaqian Zhang, KyuJung Jun, Fengyu Shen, Tofunmi Ogunfunmi, Yingzhi Sun, Michael C. Tucker, Gerbrand Ceder, Mary C. Scott

**Affiliations:** ^1^ National Center for Electron Microscopy, Molecular Foundry, Lawrence Berkeley National Laboratory, Berkeley, CA 94720, USA.; ^2^ Department of Materials Science and Engineering, University of California at Berkeley, Berkeley, CA 94720, USA.; ^3^ Materials Science Division, Lawrence Berkeley National Laboratory, Berkeley, CA 94720, USA.; ^4^ Department of Mechanical Engineering, Rochester Institute of Technology, Rochester, NY 14623, USA.; ^5^ Energy Storage and Distributed Resources Division, Lawrence Berkeley National Laboratory, Berkeley, CA 94720, USA.

## Abstract

Revealing the local structure of solid electrolytes (SEs) with electron microscopy is critical for the fundamental understanding of the performance of solid-state batteries (SSBs). However, the intrinsic structural information in the SSB can be misleading if the sample’s interactions with the electron beams are not fully understood. In this work, we systematically investigate the effect of electron beams on Al-doped lithium lanthanum zirconium oxide (LLZO) under different imaging conditions. Li metal is observed to grow directly on the clean surface of LLZO. The Li metal growth kinetics and the morphology obtained are found to be heavily influenced by the temperature, accelerating voltage, and electron beam intensity. We prove that the lithium growth is due to the LLZO delithiation activated by a positive charging effect under electron beam emission. Our results deepen the understanding of the electron beam impact on SEs and provide guidance for battery material characterization using electron microscopy.

## Teaser

The growth of Li on solid electrolytes under electron microscopy is due to the delithiation activated by a positive charging.

## INTRODUCTION

Solid-state batteries (SSBs) are the holy grail for next-generation batteries due to their potential for improved safety and energy density (*1*, *2*). However, the limited cycling life of the SSBs severely hinders their practical application (*3*–*5*). Many factors are involved in the performance degradation, including the growth of dendrites from the anode into the solid electrolyte (SE), interfacial issues (such as contact loss and chemical reactions) between the electrodes and the SE, and microstructural deterioration in the SE and electrodes during cell cycling (*6*–*10*). The development of high-performance SSBs with a more rational understanding and precise control of the underlying microstructural features requires high-resolution characterization techniques (*11*, *12*). Electron microscopy provides rich information on the structure, morphology, chemistry, and chemical composition of the battery materials at many length scales, enabling us to establish the “composition-structure-performance” triad and further guide the design of new battery materials with improved performance (*13*, *14*). Electron microscopy also offers the capability of direct and in situ observation of the electrochemical process in the batteries, for example, Li deposition at the SE/electrode interfaces and (de)lithiation within electrodes (*15*–*19*).

However, it is well known that electron beam used in transmission electron microscopy (TEM) and scanning electron microscopy (SEM) can cause temporary or permanent changes to the surface or bulk structure of a specimen through damage mechanisms such as radiolysis, knock-on displacement, diffusion, and electrostatic charging (*20*). In particular, many battery materials are extremely sensitive to electron beams (*20*–*24*). For example, researchers have shown that Li metal becomes extremely unstable under electron beam irradiation (*20*, *22*). Although some work has claimed that material characterization at a low beam intensity and low temperature can prevent the damage, many battery materials are still observed to be susceptible to electron beam damage in the TEM and SEM (*25*). Because TEM and SEM are frequently used as primary tools for structural characterization, it is important to differentiate between valid evidence originating from the electrochemical process of the SSB and invalid artifacts from beam effects during TEM/SEM characterization. For example, many previous studies have observed isolated deposition of Li inside the SE and attribute this to the nonnegligible effect of electronic conductivities of the SE (*11*, *26*, *27*). However, similar Li growth phenomena are also observed because of the electron beam effect during SEM/TEM characterization (*28*, *29*).

The growth of Li metal on the lithium lanthanum zirconium oxide (LLZO) surface under TEM/SEM has been previously observed (*23*, *28*, *29*). However, a systematic investigation of this phenomenon is missing, and the mechanism by which Li grows on the LLZO surface is still under debate. For example, Krauskopf *et al.* (*28*) observe the growth of Li on the LLZO surface and attribute it to the potential LLZO decomposition under the electron beam in the SEM. Xie *et al.* (*23*) attribute this phenomenon to electrostatics of the local electric field that develops due to the charging by the electron beams, while Liang *et al.* (*29*) argue the Li growth is due to the decomposition of a contamination layer (Li_2_CO_3_) on the LLZO surface.

In this work, we perform a systematic investigation of the interaction between electron beams and LLZO under TEM and SEM that combines in situ heating, cooling, ion-milling, and air-free transfer systems. We show that Li expulsion occurs directly on the clean surface of LLZO at both room temperature and cryogenic temperature. The Li metal growth kinetics and morphology can be controlled by changing the accelerating voltage and intensity of the electron beam. In addition, we prove that electrostatic charging is responsible for the Li growth, instead of decomposition due to irradiation. Our calculations show that the incident electron beam can induce a positive electrostatic charge, which can, in turn, result in a sufficiently high voltage (>4.3 V) to drive the delithiation reaction without damaging the primary structure of LLZO.

## RESULTS

### In situ formation and destruction of Li metal on LLZO at room temperature

**Fig. 1.**
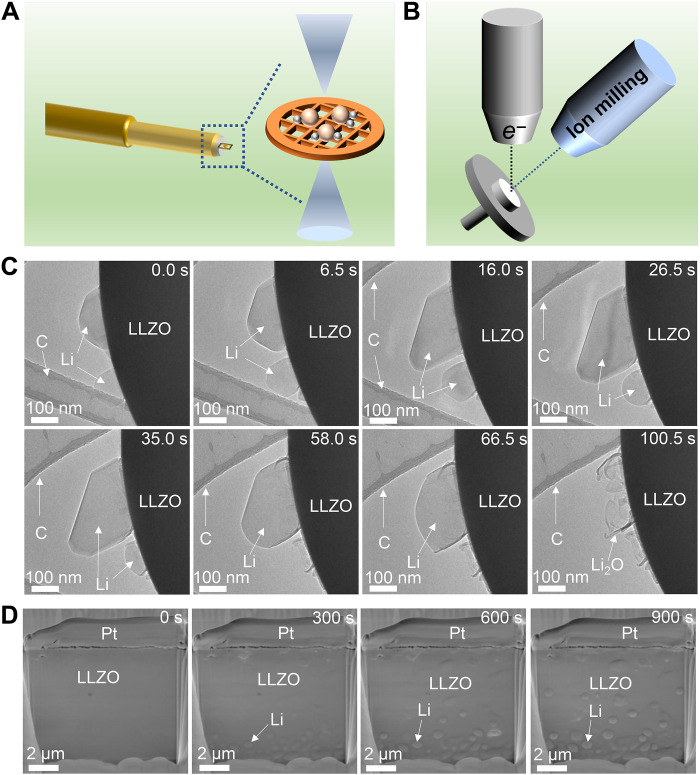
In situ formation of Li metal from LLZO at room temperature. Schematic of the experimental process of Li metal growth on the surface of LLZO under (**A**) TEM and (**B**) SEM, respectively. (**C**) Sequential TEM images showing the Li growth and annihilation on the pure surface of LLZO at an electron dose rate of 50 *e*^−^/Å^2^ · s. (**D**) Sequential SEM images showing the lithium precipitation process from a clean surface of LLZO pellet.

Figure 1 (A and B) shows the experimental setup to study the growth of Li metal on the surface of LLZO under TEM and SEM, respectively. TEM image and energy-dispersive x-ray spectroscopy (EDS) results indicate a contamination layer of Li_2_CO_3_ on the commercial LLZO particle (figs. S1 and S2), which is due to the exposure of LLZO powder to the ambient atmosphere (*30*, *31*). Li growth is observed on the surface of this Li_2_CO_3_-coated LLZO in the TEM (fig. S3), which is consistent with recently published results by Liang *et al.* (*29*). The Li_2_CO_3_ layer was eliminated by annealing in a heating holder within the electron microscopy (fig. S4). The growth of Li metal is still observed on the clean surface of LLZO at an electron dose rate of 50 *e*^−^/Å^2^ · s in the TEM, as shown in Fig. 1C. A clear “growth-destruction” behavior, with an initial period of Li particle growth, followed by erosion of the two Li nanoparticles (the gray areas in Fig. 1C) on LLZO was observed from the sequential TEM images. Notably, at the end of the destruction (*t* ≈ 100 s), the outside boundaries of the two Li particles were left behind. As will be discussed later, the leftover material is the native lithium oxide layer, which is more resistant to knock-on damage than pure Li (*20*, *32*). It is observed that most Li expulsion happens within the dose range of 20 to 300 *e*^−^/Å^2^ · s in the TEM. No obvious lithium growth is observed when the electron beam dose is too low or when it is too high. Instead, amorphization is observed in the initially crystalline structure of LLZO at high doses (fig. S5), as has been found in other similar work (*29*).

Li metal growth is further studied on a clean and freshly created LLZO surface in SEM with the results shown in Fig. 1D. The LLZO pellet was made by sintering LLZO powder (details in Materials and Methods). The cross section of the LLZO was first milled out with the Ga^+^ ion source and then polished with a low-current ion beam to minimize the surface damage until a clean and flat surface was achieved. In situ measurements indicate that some Li particles grew out from the ion-milling polished surface after minutes of electron beam irradiation at a low accelerating voltage of 5 kV. Our results show that Li metal growth can happen on a pure and clean LLZO surface in both TEM (Fig. 1C) and SEM (Fig. 1D).

### In situ TEM imaging of Li metal growth on LLZO at cryogenic temperature

**Fig. 2.**
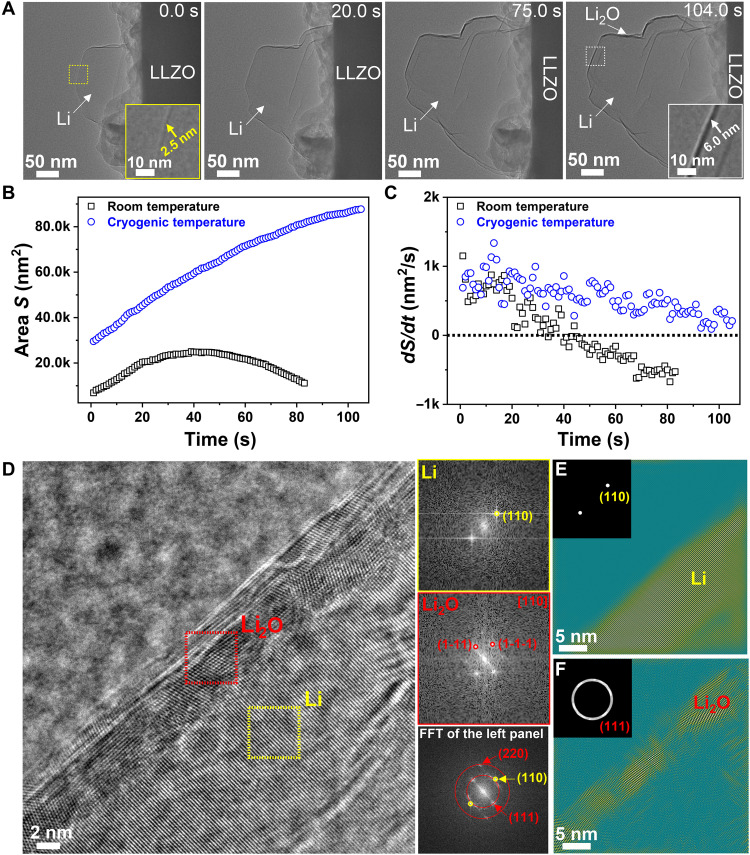
In situ TEM imaging of Li metal growth on LLZO at cryogenic temperature. (**A**) Sequential TEM images showing the Li metal growth on LLZO at cryogenic temperature. The insets are the close-up views of the yellow and white square regions, respectively. (**B**) Plots of projected areas of the representative Li nanoparticles versus time at room temperature and cryogenic temperature. (**C**) Growth/annihilation rate of the Li nanoparticles versus time at room temperature and cryogenic temperature. (**D**) High-resolution TEM (HRTEM) image of the new formed Li nanoparticle covered by an oxide layer. The corresponding FFT images of selected red region, yellow region, and the whole region are displayed from the top to bottom in the right panel. (**E**) Lithium and (**F**) lithium oxide and their corresponding selected FFT images inset, respectively.

Because electron beam damage is minimized at cryogenic temperatures (*14*, *22*, *25*, *33*), we used cryo-TEM (details in Materials and Methods) for a more detailed investigation of the Li behavior on the LLZO surface. The LLZO structure’s stability under electron beam irradiation at cryogenic temperature was studied first with a high-beam dose rate. Our in situ results (fig. S6) show that the LLZO crystalline structure is well preserved for more than 80 s at a dose rate of 3700 *e*^−^/Å^2^ · s but loses its crystallinity when the dose reaches 7740 *e*^−^/Å^2^ · s (fig. S7), consistent with our observation of LLZO amorphization at room temperature (fig. S5). When the electron dose rate is lowered to 50 *e*^−^/Å^2^ · s, the Li metal growth on the LLZO surface still occurs at cryogenic temperature, as shown in Fig. 2A.

Figure 2A shows the in situ Li growth in cryo-TEM with the same dose rate as used in Fig. 1C (50 *e*^−^/Å^2^ · s). Notably, the Li metal that grows out from the LLZO is preserved in cryo-TEM (Fig. 2A), while Li was eroded by the beam at room temperature at the same dose rate of 50 *e*^−^/Å^2^ · s (Fig. 1C). A detailed comparison of the Li metal growth kinetics at room temperature and cryogenic temperature is shown in Fig. 2B (the projected area of Li metal) and Fig. 2C (the growth rate of the projected area). During the Li growth stage at room temperature (0 < *t* < 40 s), the area of Li metal increases initially and then gradually slows down and reaches its maximum at *t* = 40 s. After that, the destruction stage starts (black line in Fig. 2B). Different from the process in which Li metal first forms and then disappears at room temperature, the Li metal keeps growing and does not erode at cryogenic temperature. The initial growth rates are the same in both cases (~10^3^ nm^2^/s) but the room temperature growth rate decreases linearly (Fig. 2C) to ~ −10^3^ nm^2^/s (Li destruction), while it reaches about zero (Li preservation) at cryogenic temperature.

During the Li growth, the initial surface layer is thin with low contrast, as indicated by the yellow arrow in the left image of Fig. 2A. After 104 s, this surface layer becomes much thicker with higher contrast, as indicated by the white arrow in the right image of Fig. 2A. Figure 2D shows the high-resolution TEM (HRTEM) images of the Li nanoparticle within the surface layer. The crystalline structure of Li metal (yellow region in Fig. 2D) and Li_2_O (red region in Fig. 2D) were determined by their corresponding fast Fourier transform (FFT) images, respectively. The inverse FFT images of the pattern in the inset of Fig. 2 (E and F) show that Li_2_O is distributed on the outer surface of the Li nanoparticle. This is consistent with previous observations that alkali metal oxidation can occur within the TEM vacuum chamber (10^−5^ Pa) at room temperature (*29*). Here, we further confirm that Li metal oxidation can even happen within the TEM at cryogenic temperature. In addition, the in situ formation of the Li_2_O layer was captured by our HRTEM results (fig. S8).

### Li growth on LLZO controlled by electron beam accelerating voltage and current within SEM

**Fig. 3.**
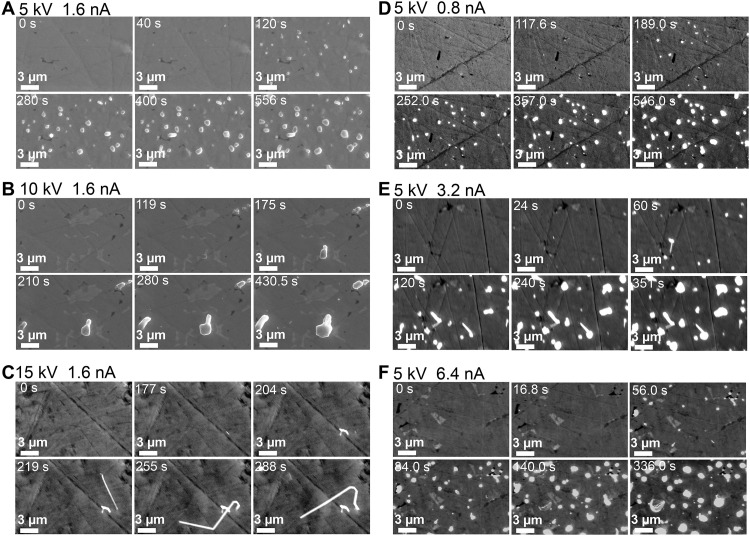
Li metal nucleation and growth on LLZO surface under in situ SEM. Image captured at a probe current of 1.6 nA and an accelerating voltage of (**A**) 5 kV, (**B**) 10 kV, and (**C**) 15 kV. Images captured at an accelerating voltage of 5 kV and a probe current of (**D**) 0.8 nA, (**E**) 3.2 nA, and (**F**) 6.4 nA.

To further understand the kinetics and mechanism of Li growth, a comprehensive in situ SEM investigation under different electron beam acceleration voltages and probe currents was conducted, as shown in Fig. 3. LLZO pellets were polished in the glove box and transferred to the SEM chamber using a commercial air-isolated transfer system to avoid air exposure. X-ray diffraction (XRD) measurements confirm the cubic structure of LLZO after polishing (fig. S9). The nucleation and growth of Li metal on LLZO at different accelerating voltages (e.g., 5,10, and 15 kV) are shown in Fig. 3 (A to C) (same probe current 1.6 nA). It is clear that the Li nucleation time increases as the voltage increases: Li nucleation starts at ~40 s under voltage 5 kV and increases to ~120 and ~180 s under 10 and 15 kV,respectively. A similar trend is observed when the accelerating voltage further increases to 20 kV (>300 s), and 25 to 30 kV (No Li is observed after 10 min). The accelerating voltage also affected the Li nucleation site and the Li growth morphology. More Li nucleation sites are found at low voltage (5 kV), which grow into spherical shapes (Fig. 3A), while less nucleation sites are observed at high voltage (15 kV) with lithium growing into whiskers (Fig. 3C).

Li nucleation and growth are also investigated at different probe currents (0.8,3.2, and 6.4 nA), as shown in Fig. 3 (D to F) (same accelerating voltage 5 kV). Shorter Li nucleation time and faster Li growth kinetics are observed when the probe current is larger. Li nucleation starts at 120 s under a current of 0.8 nA, which decreases to 24 and 17 s under currents of 3.2 and 6.4 nA, respectively. No Li nucleation was observed at very small probe current (0.1 nA) after more than 10 min.

The accelerating voltage can influence the density of Li nucleation sites and growth models, while beam current can change the growth kinetics. The Li metal growth is closely related to the emission of secondary electrons generated under the electron irradiation in SEM, a point which we return to later. When a higher probe current was used, overall more secondary electrons can escape from the surface. Our observations show that, in this scenario, growth kinetics are faster. The growth of Li metal under different accelerating voltages is more complicated. At a low accelerating voltage, the incident electrons penetrate a thin surface region, and proportionately more secondary electrons generated can escape from the surface. Therefore, we can observe many nucleation sites. However, at a high accelerating voltage, most secondary electrons are generated well below the surface region and stay inside the sample. Furthermore, the growth of lithium will change the surface properties of LLZO (such as inducing surface cracking) (fig. S10), so if a large number of secondary electrons diffuse out through the surface of LLZO and Li metal in a short time, these changes may alter the growth model of Li.

### Topotactic reaction of LLZO is the reason for Li metal growth on LLZO surface

**Fig. 4.**
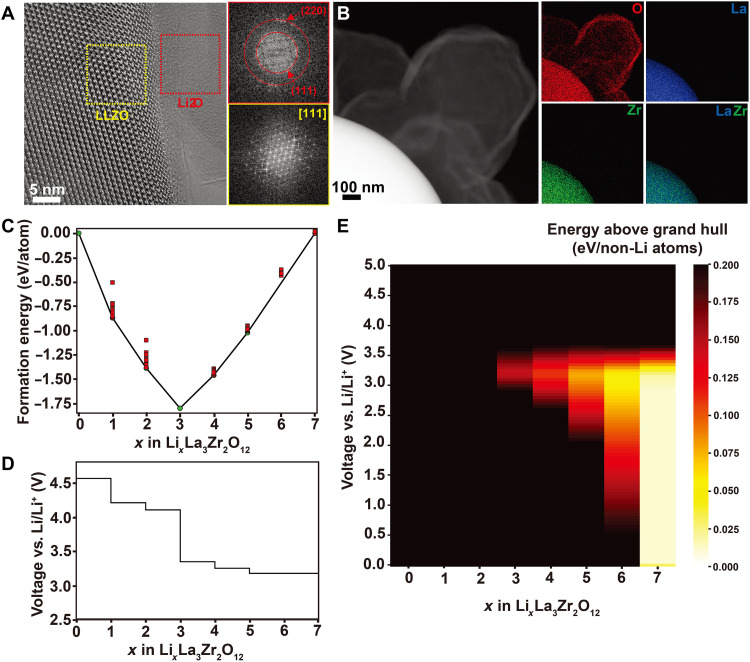
Structure characterization of LLZO after Li expulsion and topotactic reaction of LLZO during Li expulsion. (**A**) HRTEM image of LLZO surface after the growth of Li. The corresponding FFT images of selected red region (Li-related compounds) and yellow region (LLZO) are displayed in the right panel. (**B**) Representative HAADF-STEM image and the corresponding elemental maps of La, Zr, and O using Super-X EDS at cryogenic temperature. (**C**) Lithium vacancy configurations in LLZO with Li content *x* from 0 to 7. Green spheres represent configurations that are on the binary convex hull of Li_7_La_3_Zr_2_O_12_ and La_3_Zr_2_O_12_. Red spheres represent configurations that are above the binary convex hull. (**D**) Topotactic reaction potential versus Li/Li^+^. (**E**) Energy above the grand convex hull at various voltage versus Li/Li^+^ (lithium chemical potentials) and lithium content *x* in LLZO.

The fact that Li metal originates from the LLZO material raises an important question: What is the mechanism for Li formation on the LLZO surface? In principle, when exposed to a lithium chemical potential beyond its intrinsic stability window, any lithium-containing material is expected to undergo a thermodynamically intrinsic decomposition pathway, resulting in the formation of ground-state decomposition products. However, there can be various other metastable reaction pathways that have smaller driving force but may have significantly lower kinetic barrier. A good example of a metastable reaction pathway is the topotactic lithium insertion and extraction reaction (*34*, *35*). As the topotactic reaction maintains the structural framework and does not require the formation of a new crystalline decomposition product, it is expected to have much faster kinetics than the intrinsic decomposition reactions. With LLZO being a superionic conductor exhibiting lithium ionic conductivity over 0.1 mS/cm for a relatively wide range of lithium content 6 < *x* < 7 (*36*), it is reasonable to expect that lithium extraction can proceed with a low kinetic barrier. Earlier work argued that the Li growth kinetics are related to the decomposition of Li compounds in the surface area subject to electron beam irradiation (*28*). Density functional theory (DFT) calculations also show that decomposition can happen when the oxidation potential is reached (*34*). If the decomposition reaction indeed happens, then the surface structure of LLZO cannot be preserved and decomposition products should be observed. However, our HRTEM and EDS mapping results shown in Fig. 4 disprove this structural change or the appearance of a new structure.

Figure 4A displays the HRTEM images of the LLZO after Li metal formation. As shown in Fig. 2, Li metal oxidation can still occur when the sample is cooled to a cryogenic temperature, and the thickness of the Li_2_O layer increases during the Li metal formation process. In addition, the contrast of the HRTEM image of Li on the carbon film is low, which makes it hard to distinguish in the images. Similarly, the Li has much less contrast than the LLZO so that only Li particles that extend past the edge of the LLZO particles can be observed. Compared with Li_2_O, the stability of Li metal under the irradiation of the electron beam is much worse. Although the low temperature can reduce the beam effect to some extent, the Li metal still will still quickly disappear in the process of imaging at high magnification (fig. S11), making the Li_2_O much easier to observe than Li. The selected yellow region shows that the LLZO crystalline structure is maintained, as indicated in the well-matched FFT image along [111] direction. The high-angle annular dark-field scanning TEM (HAADF-STEM) indicates a low-contrast region between two bright LLZO particles (left image of Fig. 4B), which is further shown to contain an oxide shell (Li_2_O) as indicated by the O region in the EDS images in Fig. 4B. The uniformly distributed La and Zr elements further confirm that the LLZO structure is preserved.

The absence of any decomposition products of LLZO in TEM images and the fact that the crystal structure of LLZO remains intact leave a topotactic reaction of LLZO as the only possible mechanism for Li expulsion. Previous work observed that the SE material itself can undergo a metastable reaction pathway that incorporates redox activities by topotactic lithium insertion and extraction (*34*, *35*), resembling typical intercalation electrode materials. To elucidate the competition between topotactic delithiation and decomposition of LLZO at various lithium chemical potentials, we compute the formation energies of various lithium vacancy configurations at different lithium concentrations *x* of Li*_x_*La_3_Zr_2_O_12_ (details in Materials and Methods). Figure 4C shows the convex hull of the formation energies of topotactic delithiation reaction from the LLZO framework, and Fig. 4D shows its corresponding voltage profiles. Our calculations indicate that from *x* = 7 to 3, lithium can be extracted at ≈3.2 V versus Li metal. Reaching a lithium content below *x* = 3 requires a higher voltage of ≈ 4.2 V.

However, as lithium extraction reaction occurs by the oxidation of O^2−^ anions, the framework will become highly metastable as lithium is removed (fig. S12), making the decomposition reaction more and more favorable. Figure 4E illustrates the grand potential energy of topotactically delithiated phases of LLZO embedded in a variety of lithium chemical potentials. At its pristine state, LLZO lies slightly above the of grand canonical convex hull (0.011 eV/atom) throughout a wide range of lithium chemical potentials from 0.046 to 2.9 V, which is in excellent agreement with the previously reported electrochemical stability window of LLZO (*24*, *37*). However, in delithiated LLZO of *x* = 6, the minimum energy above the grand canonical convex hull is 0.052 eV/atom at 3.16 V and remains below 0.1 eV/atom between 2.25 and 3.35 V. As more lithium is extracted to *x* = 3, the minimum energy above the convex hull of grand potential energies increases, and the range of lithium chemical potentials at which the energy above hull remains below 0.1 eV/atom quickly narrows. Last, as LLZO is further extracted below *x* = 3, delithiated LLZO becomes highly unstable in any lithium chemical potentials, with energy above the hull always larger than 0.1 eV/atom.

As the driving force for decomposition increases as more lithium is extracted from LLZO, topotactic delithiation is expected to stop at some value *x*_topotactic limit_ at which the decomposition reactions will take over. Our analysis indicates that when lithium is topotactically extracted down *x* = 6 the metastability remains within a reasonable range around 0.052 eV/atom. Further delithiation to *x* = 3 may occur, after which the decomposition energy becomes so large that further topotactic delithiation is unlikely and decomposition should set in. The exact topotactic delithiation limit could be determined in principle by careful electrochemical measurements.

### The voltage for topotactic reaction is from a positive charging effect

It should be noted that a reducing environment is expected in the LLZO sample under electron beam injection while an oxidizing potential higher than ~3.2 V (as discussed in the previous section) is required for the topotactic reaction to take place. The apparent contradiction is reconciled by recognizing that the incident electron beam can create regions of positive charge within insulators. The charging of an electrically resistive sample (such as LLZO) occurs when the specimen has an excess or a lack of electrons due to the generation and emission of secondary electrons (*38*, *39*). A steady-state current balance equation below can be used to qualitatively describe the charging effect

$$I_{0}+\frac{V_{s}}{R_{s}}=I_{0}\eta +I_{0}\delta +I_{t}$$ (1)

Terms on the left-hand side represent current from the incident beam (*I*_0_) and the leakage ($\frac{V_{s}}{R_{s}}$), with *V*_s_ being the surface potential of LLZO sample and *R*_s_ being the effective electrical resistance between the irradiated and surrounding regions of LLZO. Terms on the right-hand side represent the loss of electrons by backscattering (*I*_0_η) with η being the backscattering coefficient, secondary emission (*I*_0_δ) with δ being the yield for secondary electrons, and the transmitted current (*I*_t_). The surface potential *V*_s_ can be negative or positive depending on the incident beam voltage (*E*_0_), the incident current *I*_0_, and the LLZO properties (such as the density, plasmon energy, and bandgap) (*40*).

When a thick LLZO sample is used in the SEM, no electrons can penetrate through the sample, and therefore, the transmitted current *I*_t_ is zero. At low *E*_0_ in SEM, the incident electrons penetrate only a few nanometers (or less), and most of the secondary electrons generated can escape into the vacuum. The larger value of secondary emission (*I*_0_δ) requires the positive surface voltage *V*_s_ to be developed to reach current balance in Eq. 1. The further rising of the positive *V*_s_ makes it harder for secondary electrons to leave the sample surface and therefore δ decreases. This process continues until an equilibrium state is reached, with the surface potential *V*_s_ stabilized at a positive value. This positive potential serves as the oxidation potential required by the LLZO topotactic reaction for Li metal growth as discussed in the SEM section. However, at high *E*_0_ in SEM, most secondary electrons are generated well below the sample surface and can only stay inside the sample. Consequently, the secondary emission (*I*_0_δ) is low and the surface potential *V*_s_ becomes negative. The value of *V*_s_ will be more negative when higher *E*_0_ is applied in the SEM, but this is not the case in the TEM. For the thin LLZO sample used in the TEM, a transmitted current *I*_t_ is generated because a large amount of electrons are transmitted through the sample. The transmitted current *I*_t_ can reach the magnitude of the incident current (*I*_0_) when the beam voltage (*E*_0_) is high enough, which leads to the relation: $\frac{V_{s}}{R_{s}}\approx I_{0}\eta +I_{0}\delta$. Consequently, the surface voltage *V*_s_ will be positive again when *E*_0_ is higher than a critical value. This positive *V*_s_ serves as the oxidation potential required for the topotactic Li extraction from LLZO as shown in the TEM.

**Fig. 5.**
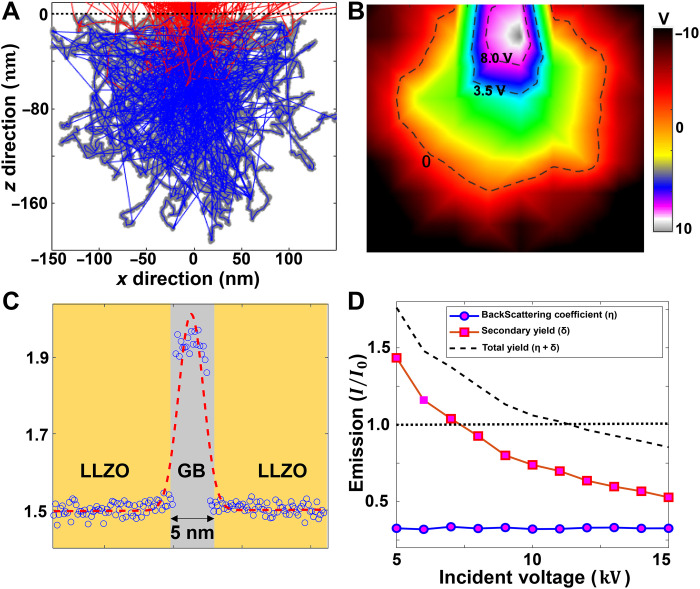
Simulation of charging effect in LLZO under electron microscopy. (**A**) Trajectories of primary electrons (blue lines) and secondary electrons that left the sample (red lines) and stayed in the sample (gray dots surrounding blue lines) at incident voltage of 5 kV. (**B**) Distribution of electric field in LLZO near the beam area, with the positive potential area highlighted by the contour lines. (**C**) Secondary electron emission (SE yield) near the grain boundary (GB) of the LLZO. (**D**) Electron emission (backscattered electron emission in blue curve and secondary electron emission in red curve) as a function of incident voltage.

A more detailed numerical simulation for the SEM case was conducted to quantitatively analyze the experimentally observed results in the SEM. Figure 5 shows the simulations of high-energy electrons (beam electrons) and their trajectories in LLZO obtained with the CASINO software (*40*). The electron trajectories (Fig. 5A) and the electric field in the LLZO sample near the beam area (Fig. 5B) are obtained from an LLZO sample with size 1 μm by 1 μm by 1 μm under an incident beam voltage *E*_0_ = 5 keV. A considerable amount of secondary electrons (gray dots surrounding blue lines in Fig. 5A) will be generated within the trajectory of the primary electrons (blue lines). However, most of these secondary electrons can only travel a very short distance due to loss of kinetic energy through multiple inelastic scattering events, while only a small portion (~0.2%) near the surface can escape from the sample, as indicated by the red lines in Fig. 5A. Figure 5C shows the yield of secondary electrons (δ) along the grain boundary (GB) (with GB thickness of 5 nm] of the LLZO, which has much smaller bandgap (~3 eV) than that of bulk LLZO (~5 eV) (*41*). Figure 5D shows the averaged electron emission (including backscattering electrons and secondary electrons) at different incident beam voltage *E*_0_.

The ratio of backscattered electrons to injected electrons (η = 0.33) and the secondary electrons to injected electrons (δ = 1.43) shown in Fig. 5D led to the result that more electrons are emitted (1.76 *I*_0_) than injected (*I*_0_). This electron deficiency in the LLZO sample may cause an electric field gradient near the surface area and induce a positive potential. The relation between the potential and charge density near the surface is described by the Poisson equation (*42*), the numerical solutions of which are displayed in Fig. 5B. The potential field ranges in magnitude from −10 to 10 V within the LLZO sample. Only those areas that satisfy the positive potential requirement (*V*_s_ > 3.5 V) can have the formation of metallic Li. These positive-potential areas are even more prevalent near GBs, as shown in Fig. 5C because the lower bandgap of LLZO near the GBs (*11*) makes secondary electrons easier to escape from the LLZO surface. The electron deficiency will disappear as the incident electron voltage increases, as shown in Fig. 5D. The total number of emitted electrons is less than the injected electrons (the *I*/*I*_0_ ratio decreases below 1) when the applied voltage is larger than 12 keV.

## DISCUSSION

We show in this work that Li metal can grow from a clean surface of LLZO in TEM/SEM at both room temperature and cryogenic temperature due to electron beam charging. The Li metal growth on the surface of LLZO occurred under electron beam irradiation (Fig. 1C and fig. S13) at different accelerating voltages (80, 200, and 300 kV). By comparing the room temperature and cryogenic in situ TEM results, we conclude that the Li growth-destruction behavior observed at room temperature is caused by beam damage, which sputters the Li particles away as their growth rate slows. At cryogenic temperatures, where beam damage is reduced, we only observe Li particle growth. Therefore, the symmetric growth-destruction behavior of Li at room temperature is a consequence of the competition between the Li nanocrystal expulsion and the Li mass loss due to knock-on beam damage. It should also be noted in Fig. 2A that the surface of the Li nanocrystal (the part in contact with the environment) remains geometrically unchanged during the Li growth (fig. S14). This indicates a root-growth mode of the Li growth kinetics: through Li deposition at the contact interface between the lower end of the Li nanocrystal and the LLZO substrate (*43*). In addition, the outer surface of expelled Li constantly changes morphology when growing at room temperature, while somewhat maintaining its shape at cryogenic temperature (Fig. 2A). This is likely due to the fast Li surface diffusion at room temperature due to the knock-on collisions and beam heating. Suppression of this diffusion at cryogenic temperature may lead to a more stable surface.

The growth of Li on the clean LLZO surface seems to contradict earlier work that concluded that the Li growth arises from the Li_2_CO_3_ contamination layer on LLZO (*29*). The apparent disagreement is due to the effect of accelerating voltage and dose rate. Liang *et al.* found that there is no Li metal growth on the clean surface of LLZO, and the surface of LLZO becomes amorphous under beam irradiation. Our results indicate that the growth of lithium depends on the electron beam dose rate used for imaging in a TEM. The Li metal growth on the clean surface of LLZO was mostly observed within a narrow electron beam dose rate range of 20 to 300 *e*^−^/Å^2^ · s at 300 kV. When the dose rate is below that, no Li metal and structure damage are observed. When the dose rate is high (e.g., 7740 *e*^−^/Å^2^ · s), we confirmed a change of the LLZO structure from crystalline to amorphous and did not observe Li metal growth.

In summary, we provide insight into electron beam charging affecting solid-state electrolyte characterization. Our observations show that Li metal can grow on a clean surface of LLZO and be oxidized at low dose and low temperature in an electron microscope. The Li expulsion from the LLZO surface is related to the internal electric field formed by the electron injection, not due to the electron beam–assisted decomposition reaction of LLZO. Furthermore, we show that the Li metal particle growth can be controlled by the beam intensity and accelerating voltage. Our findings provide detailed information on beam effects on battery materials during characterization using electron microscopy, especially the impact of beam charging.

## MATERIALS AND METHODS

### Materials synthesis

Commercial Li_6.25_Al_0.25_La_3_Zr_2_O_12_ (LLZO) powder (Ampcera, USA) was used in this study. LLZO pellets were synthesized using the following procedures. Briefly, 5 g of LLZO, 0.1 g of Li_2_CO_3_, and 0.1 g of MgO were mixed with isopropyl alcohol and ZrO_2_ balls by a jar mill. A total of 0.15 g of polyvinyl butyral was added as a binder. MgO acts as a sintering homogenizer to limit the LLZO grain growth. After overnight mixing, the slurry was dried at 70°C, and the mixed powder was separated by a sieve. The powder was pressed into pellets with a 12.7 mmh-diameter die with 400-MPa pressure. Pellets were debonded at 700°C for 1 hour in air and then sintered at 1120°C for 5 hours under argon atmosphere with heating and cooling rate of 5°C/min. Graphite sheets were placed between the pellets and Al_2_O_3_ substrates during sintering to prevent reaction. No LLZO mother powder was used to bury the pellets during sintering. Sintered pellets were polished with 600, 800, and 1200 grit silicon carbide sandpapers sequentially (Allied High Products Inc.).

### Material characterization

#### TEM characterizations

We performed HRTEM, HAADF-STEM, and EDS for LLZO using aberration-corrected electron microscopy (TEAM 1 and ThemIS) operated at 80 to 300 kV at the National Center for Electron Microscopy (NCEM), part of the Molecular Foundry at Lawrence Berkeley National Laboratory. Most of the TEM experiments were conducted at 300 kV at room temperature unless otherwise specified. The sample characterization at cryogenic temperature was achieved using a Gatan 915 cryo-holder. The LLZO powder was first dispersed on the Cu TEM grid (ultrathin carbon film on lacey carbon support film, Ted Pella Inc.) and then annealed at 750°C for 1 hour to eliminate the contamination layer within TEM using a Gatan heating holder.

To remove the contamination layer for cryo-TEM experiments, commercial LLZO powder (Amperca, USA) was pressed into pellets with a 12.7 mm-diameter die with 400-MPa pressure. Pellets were sintered at 1120°C for 5 hours under argon atmosphere. To minimize the surface contamination of LLZO upon air exposure, the LLZO pellet after sintering was polished and crushed into powder in the glove box. The powder was dispersed on a TEM grid, and then, the TEM grid was loaded on the cryo-holder in the glove box. The sample was transferred under the protection of the tip cover, holder shield, and a plastic bag and then quickly inserted into electron microscopy chamber. After that, the liquid nitrogen was poured into the dewar of the holder. The tip cover was opened for imaging the sample when the holder became stable (1.5 hours after adding liquid nitrogen).

The in situ Li metal growth was performed using the ThemIS microscope at NCEM. The nucleation and growth of Li metal on the surface of LLZO are random, and the nucleation starts within seconds under the irradiation of the electron beam. During the imaging process of the in situ study, it is challenging to capture the nucleation process due to the limited viewing area under TEM. Therefore, recording only began when a nucleation event occurred in the field of view. Thus, time = 0.0 s in all TEM images is the moment that we started to capture the growth process of Li metal and not the beginning of the Li expulsion reaction.

#### SEM characterizations

The in situ SEM study of Li metal growth was performed using FEI Helios G4 at NCEM. The LLZO pellets were first polished using the sanding paper in the glove box and then transferred to the SEM chamber using a commercial air-free transfer system (Kammrath & Weiss Transfer Module). The clean surface of the cross section of the LLZO pellet was prepared using Ga^+^ ion beam milling. The pellet was tilled to 52° and milled a hole with a beam current of 0.9 to 6.5 nA. Later, the surface was polished with 28- to 96-pA ion beam sequentially.

#### X-ray diffraction

The phase analysis was performed by XRD (D2 Phaser, Bruker) with Cu Kα1 radiation, and the test condition was 30 kV and 30 mA. The scan speed was 5°C/min within the range of 10° to 70°.

### Computational methods

#### First-principles calculations of LLZO

DFT calculations within the projector augmented wave formalism were performed (*44*), as implemented in the Vienna Ab initio Simulation Package (*45*). We used a mixed scheme combining the generalized gradient approximation (GGA) with GGA with a Hubbard correction, as proposed by Jain *et al.* (*46*). Each calculation was performed with a *k*-point grid of at least 1000/(number of atoms) and an energy cutoff of 520 eV to maintain compatibility with the Materials Project database in constructing phase diagrams (*47*).

### Thermodynamic stability analysis of LLZO delithiation

#### Grand canonical reaction energy calculations

Reaction energies of LLZO following topotactic delithiation reaction and decomposition reaction pathways were computed from the grand canonical phase diagram at various lithium chemical potentials. For a given lithium chemical potential μ_Li_, we consider the grand potential ∅ of a compound following Eq. 2, where *c* is the composition of the compound, *E*[*c*] is the enthalpy, and *n*_Li_[*c*] is the lithium concentration at composition *c* (*48*)

$$\emptyset [c,\mu_{\mathrm{Li}}]=E[c]-n_{\mathrm{Li}}[c]\mu_{\mathrm{Li}}$$ (2)

To compute the delithiation voltage profile and decomposition reaction potentials, lithium chemical potentials were converted to reaction voltage referenced by lithium metal, following Eq. 3 (*49*)

$$V_{\mathrm{reaction}}=\frac{\left(\mu_{\mathrm{Li}}^{0}-\mu_{\mathrm{reaction}}\right)}{e}\;(V)$$ (3)

For each delithiated LLZO with lithium content *x*, we computed its energy above the grand convex hull at lithium chemical potentials corresponding to voltages from 0 to 5 V, resulting in Fig. 4E.
