# Supplementary material for: Unraveling Li growth kinetics in solid electrolytes due to electron beam charging
Source: Sci Adv. 2023 Apr 26;9(17):eabq3285. doi: 10.1126/sciadv.abq3285 (PMC10132747; doi:10.1126/sciadv.abq3285)
Supplement: Supplementary file 1 — Figs. S1 to S14 [file sciadv.abq3285_sm.pdf]

Science Advances
AAAS

Supplementary Materials for

Unraveling Li growth kinetics in solid electrolytes due to electron beam charging

Xinxing Peng *et al.*

Corresponding author: Mary C. Scott, mary.scott@berkeley.edu; Qingsong Tu, howard.tu@rit.edu

Sci. Adv. **9**, eabq3285 (2023)
DOI: 10.1126/sciadv.abq3285

This PDF file includes:

Figs. S1 to S14

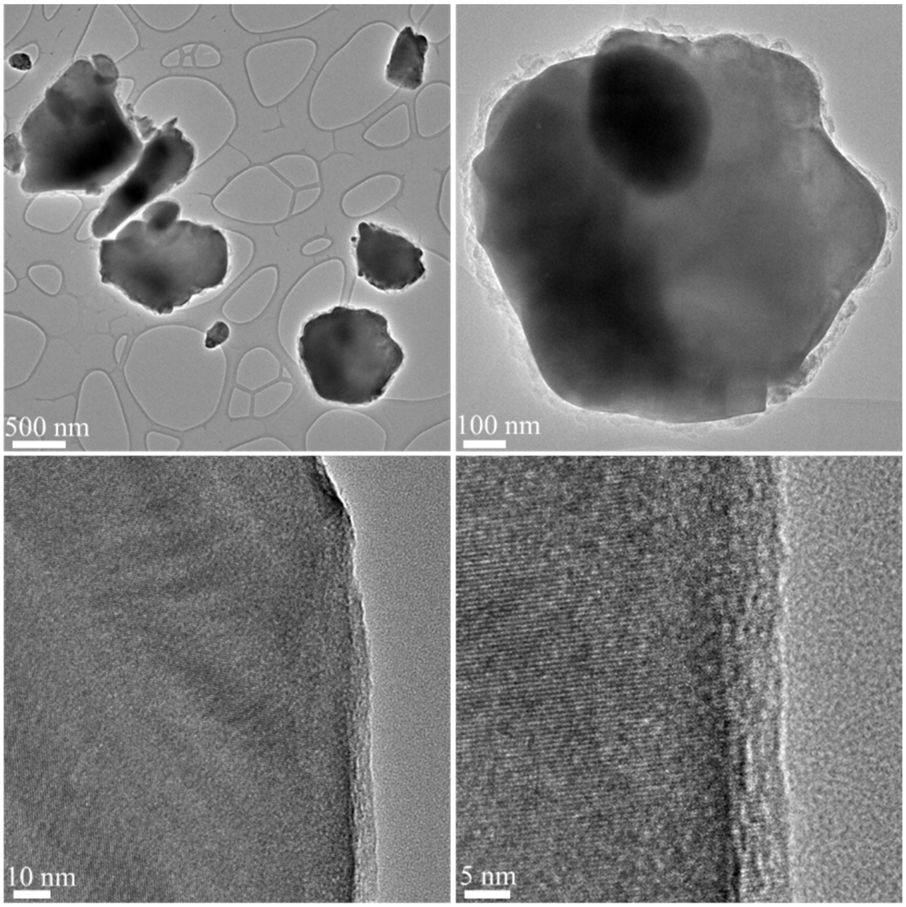

Fig. S1. Representative TEM images of commercial LLZO powder exposed to air for one week. A contamination layer was observed on the surface of LLZO.

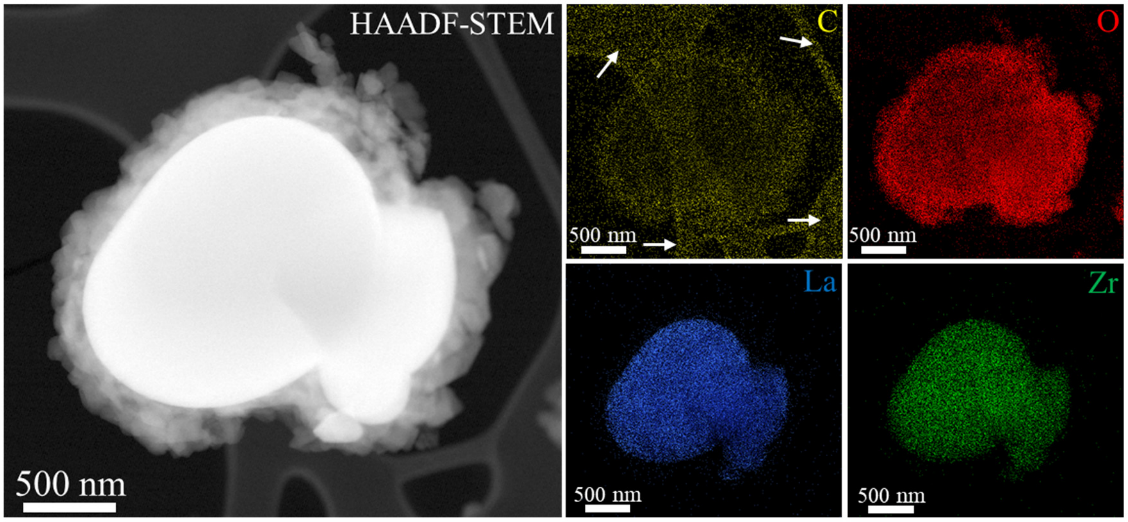

Fig. S2. Composition characterization of LLZO after air-exposure. Representative HAADF-STEM image and the corresponding elemental maps of C, O, La and Zr using super-X EDS. The carbon signal (indicated by white arrows) around the LLZO particle come forms the background of the lacey carbon film.

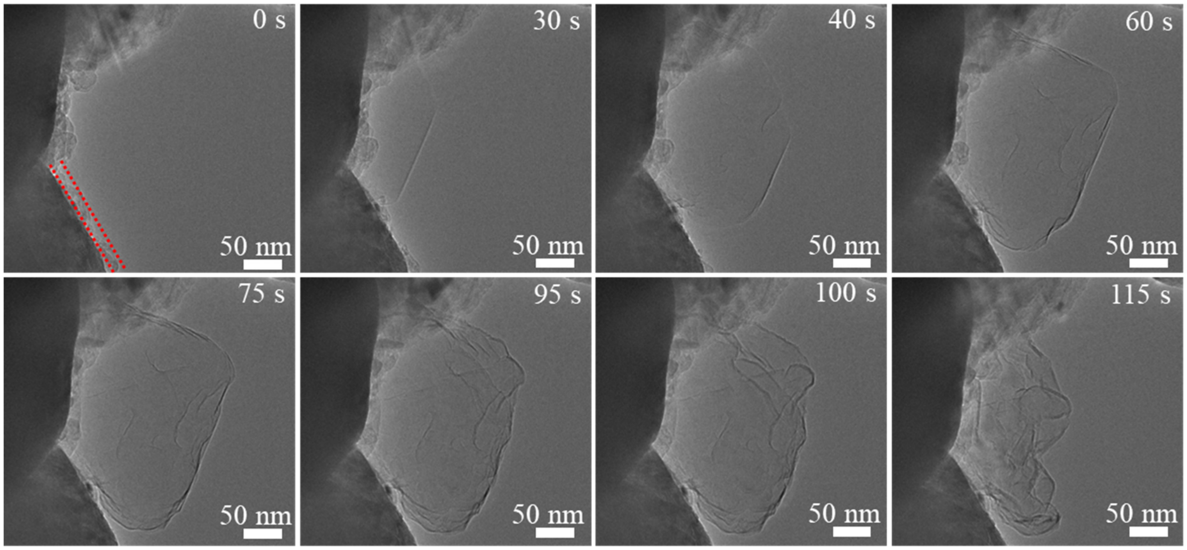

Fig. S3. Sequential TEM images showing the Li-metal growth on Li_2CO_3-coated LLZO. A contamination layer was clearly observed within the red dash line. The growth and annihilation process of Li-metal from the surface of LLZO was displayed.

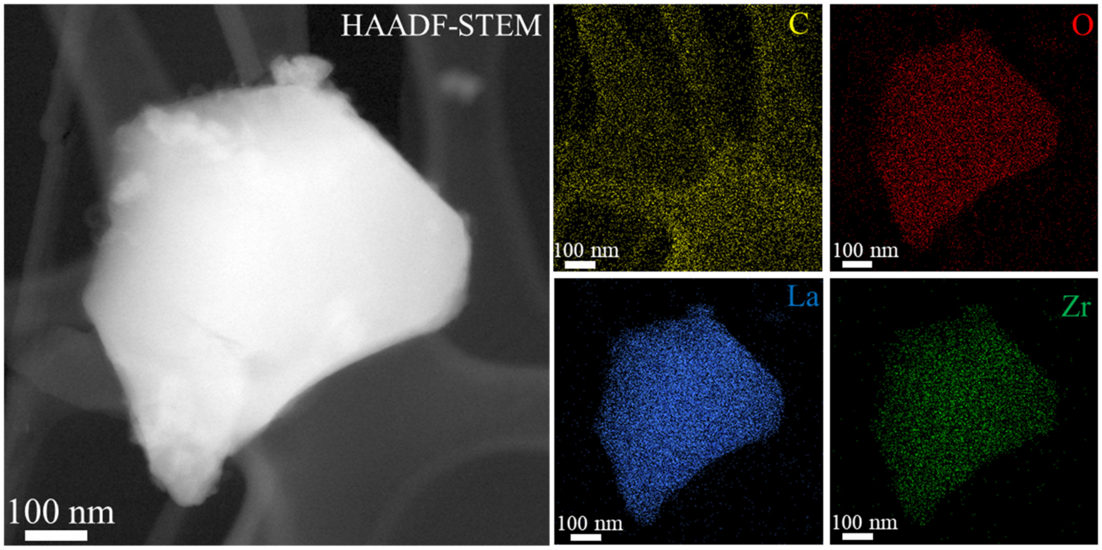

Fig. S4. Composition characterization of LLZO after removing Li_2CO_3 layer by heating. Representative HAADF-STEM image and the corresponding elemental maps of C, O, La and Zr using super-X EDS.

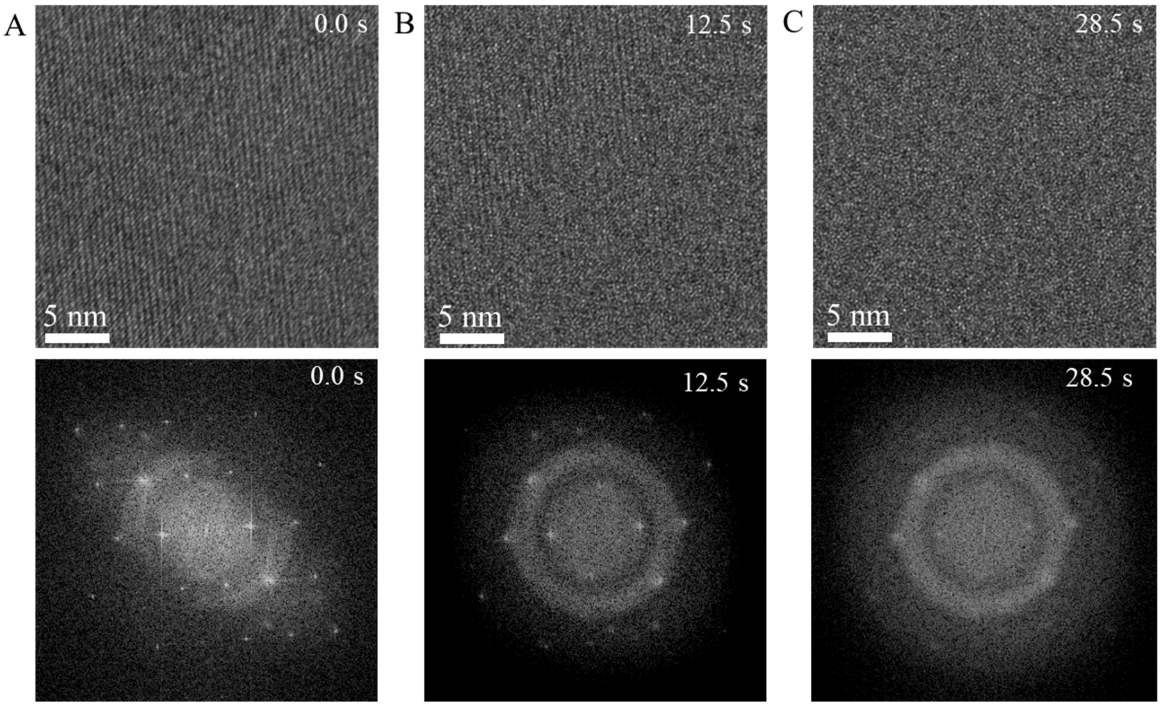

Fig. S5. Sequential TEM images showing the amorphization process of crystalline LLZO at a dose rate of 3700 $e^{-}/\text{Å}^{2}\cdot s$ under electron beam irradiation at room temperature. The TEM images and their corresponding FFT images at (A) 0.0 s, (B) 12.5 s and (C) 28.5s are displayed. Phase change caused by beam damage (crystal to amorphous) occurs within half a minute.

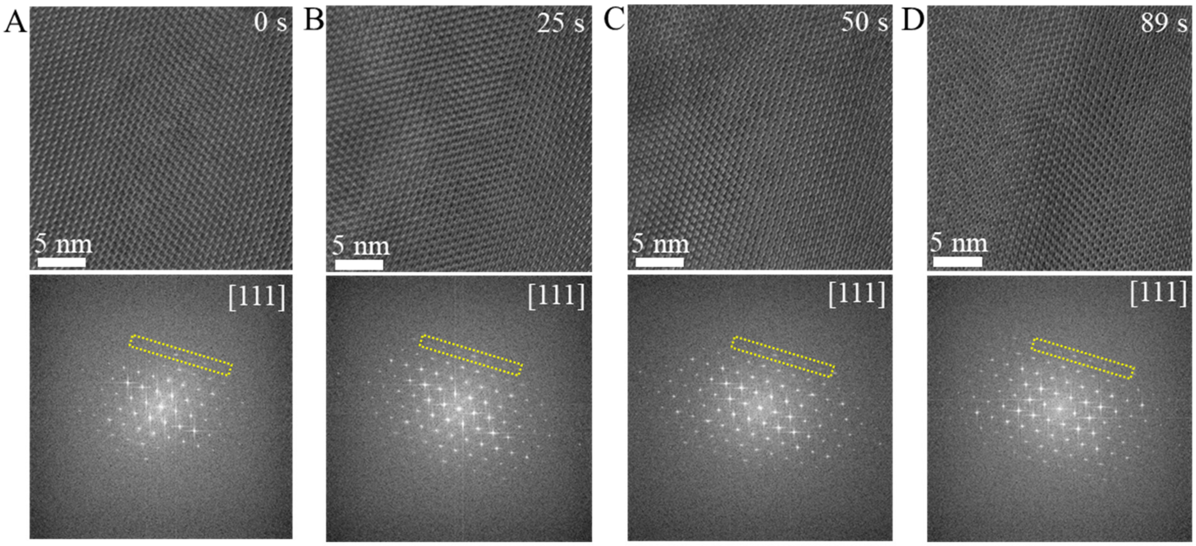

Fig. S6. Sequential TEM images showing the LLZO structure could be well preserved at a dose rate of 3700 $e^{-}/\text{Å}^{2} \cdot s$ under beam irradiation at cryogenic temperature. The TEM images and their corresponding FFT images at (A) 0 s, (B) 25 s, (C) 50 s and 89 s are displayed. The yellow dash square indicates that the crystalline structure was not damaged by high dose electron beam.

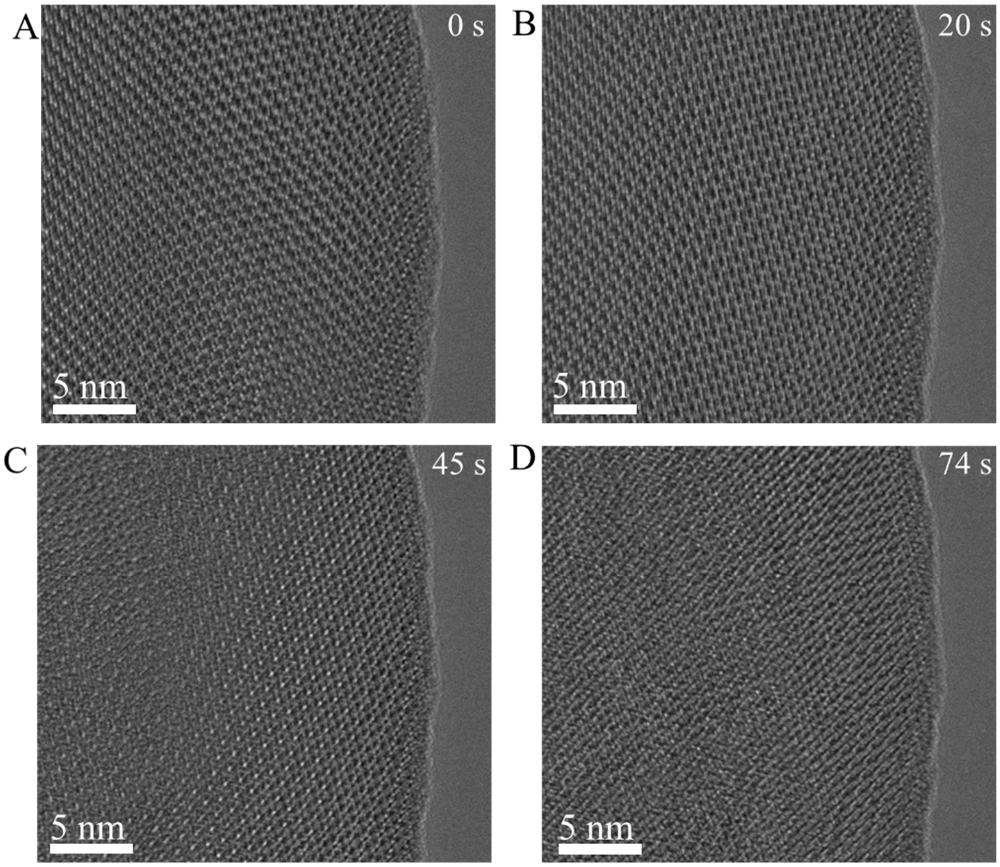

Fig. S7. Sequential TEM images showing the amorphization process of crystalline LLZO at a dose rate of 7740 $e^{-}/Å^{2} \cdot s$ under electron beam irradiation at cryogenic temperature. The TEM images at (A) 0 s, (B) 20 s, (C) 45 s and 74 s are displayed.

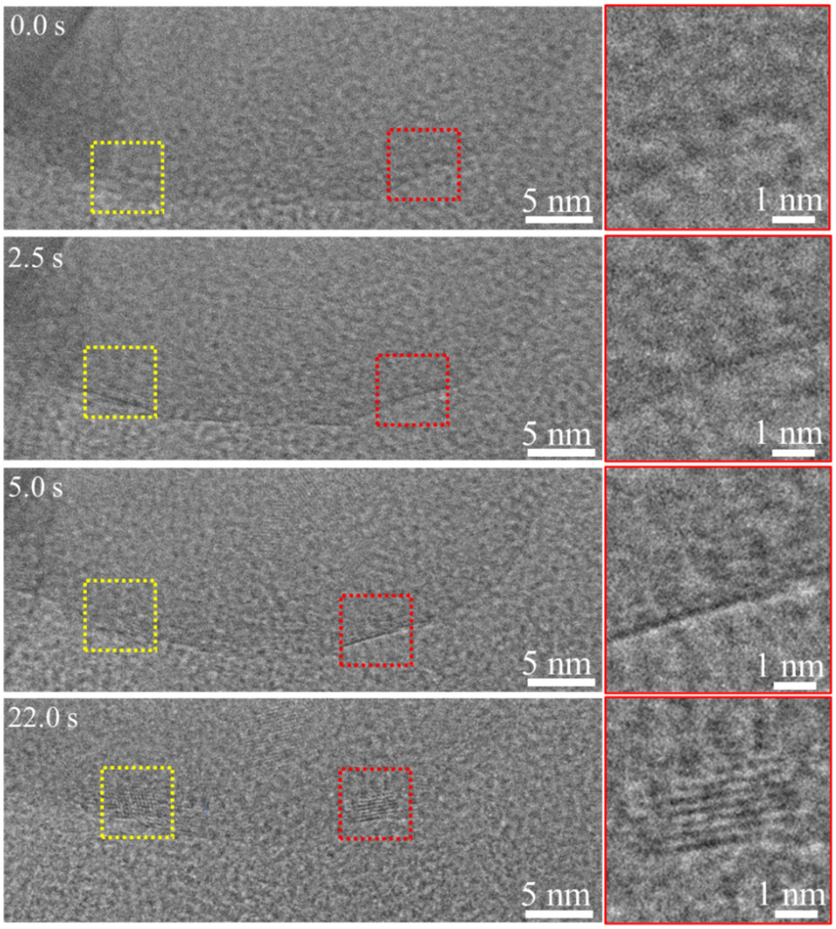

Fig. S8. Sequential HRTEM images showing the initial surface oxidation layer formation. The red and yellow square regions highlight the surface oxidation layer formation. The right images are the enlarge regions of the red square in the left images.

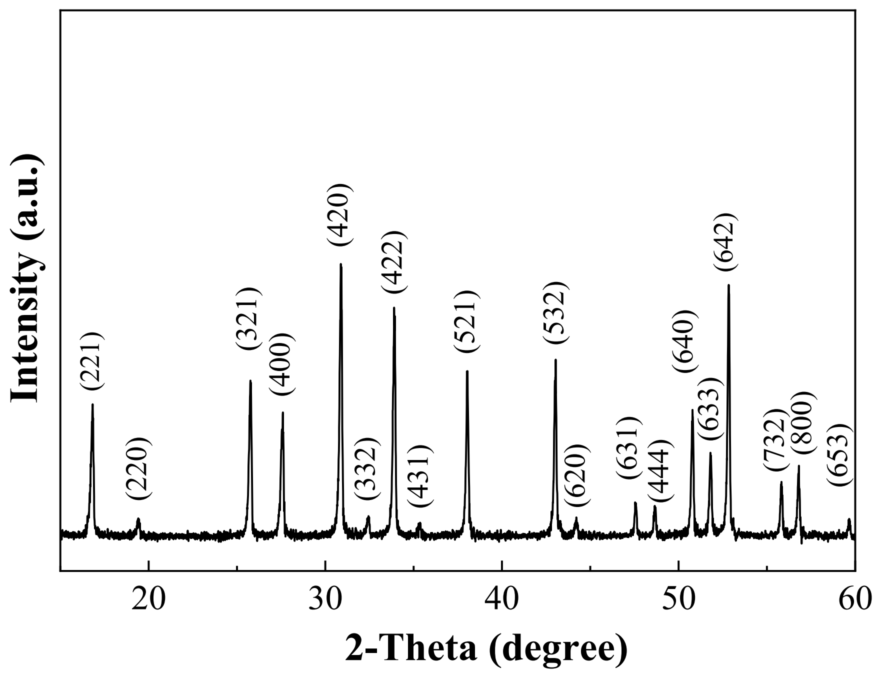

Fig. S9. XRD pattern of the LLZO pellet after polishing.

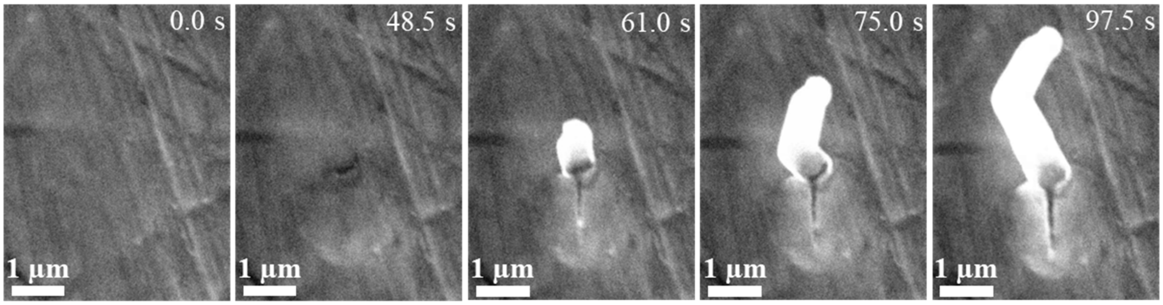

Fig. S10. Sequential SEM images showing the formation of surface crack during Li growth.

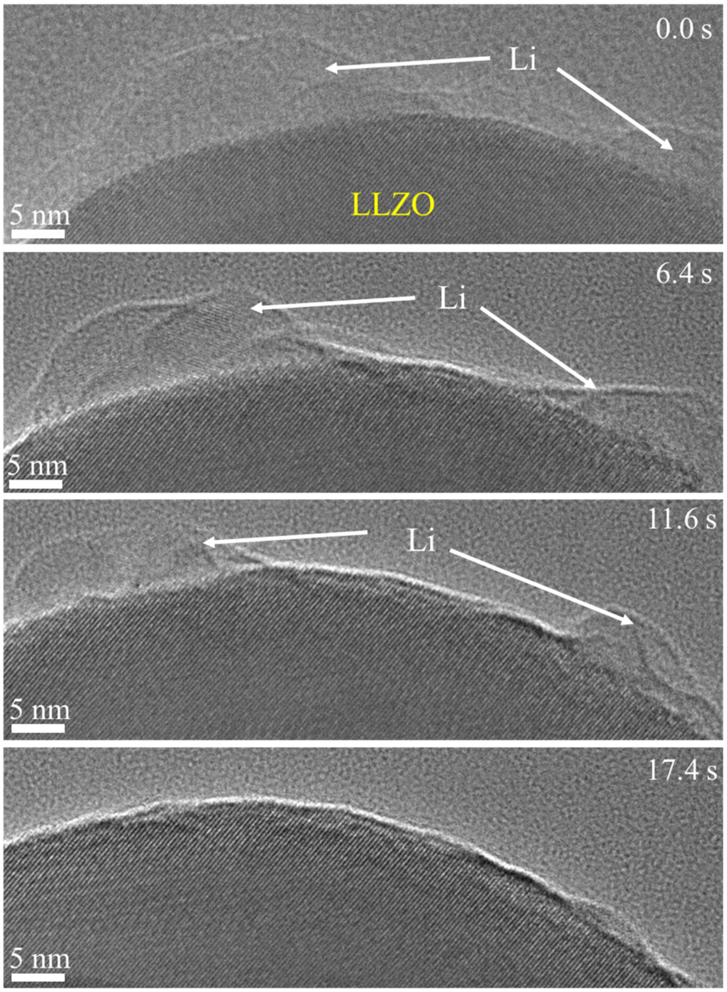

Fig. S11. Sequential TEM images showing the disappearance of Li at cryogenic temperature.

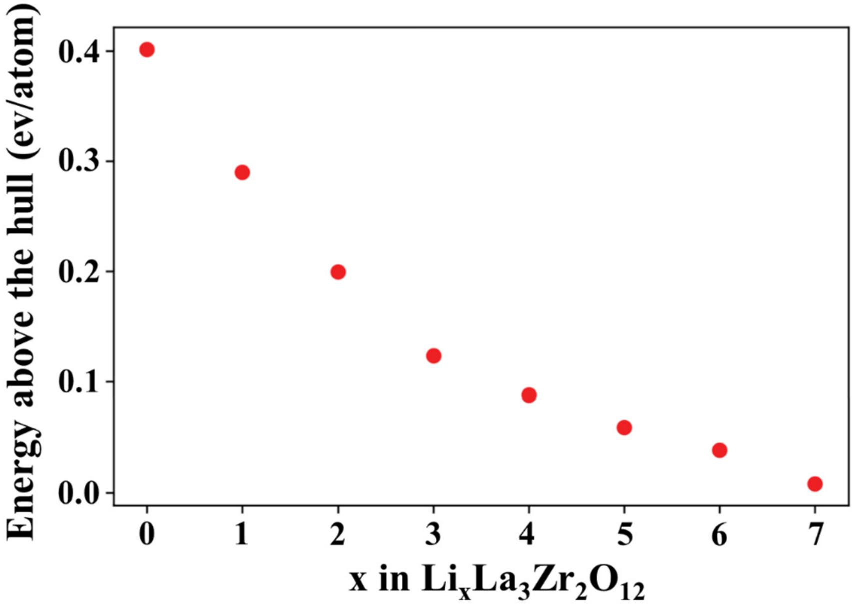

Fig. S12. Energy above the hull *vs* the lithium content *x* in LLZO. The framework becomes highly metastable with lithium extraction, and decomposition reaction becomes more favored as more lithium is extracted.

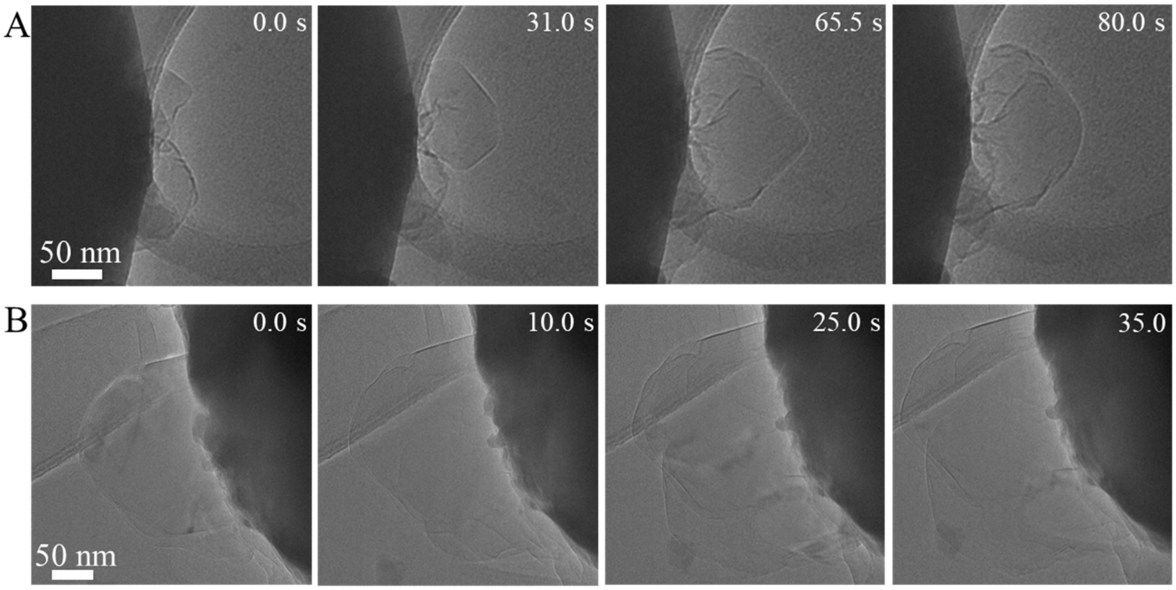

Fig. S13. Sequential TEM images showing the Li-metal growth on LLZO at 80 kV and 200 kV. The growth of Li-metal on the surface of LLZO at 80 kV (A) and 200 kV (B) at a dose rate of 50 $e^{-}/\text{Å}^{2} \cdot s$.

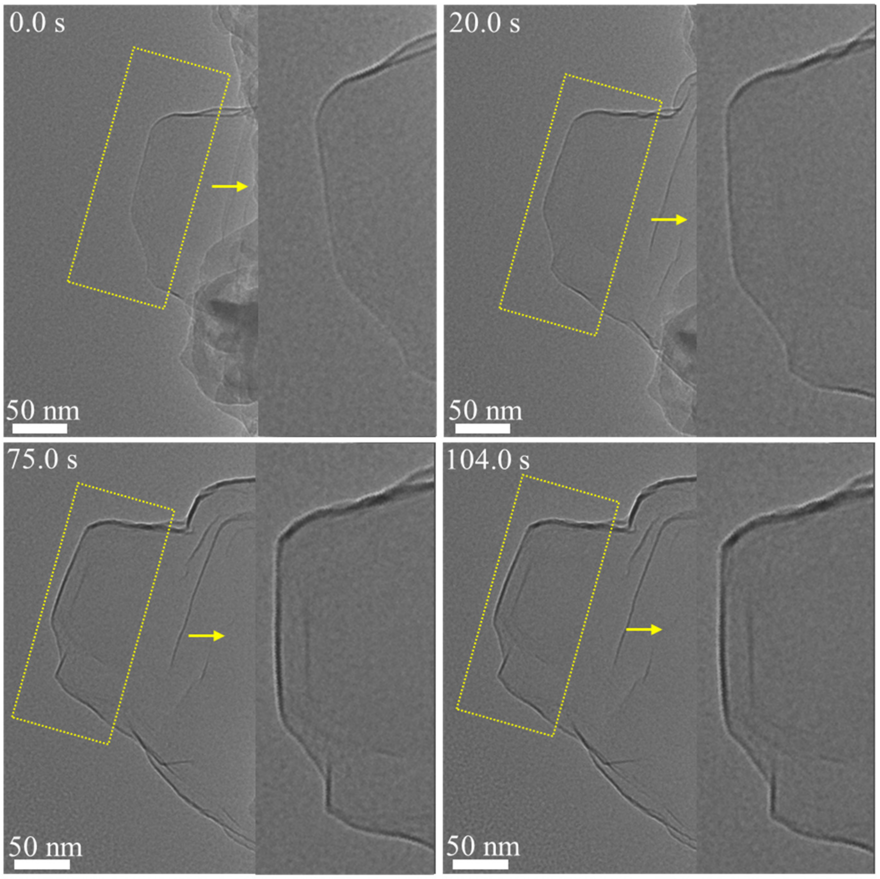

Fig. S14. Sequential TEM images showing the Li-metal growth on LLZO at cryogenic temperature. The surface of Li nanocrystal remains geometrically unchanged during the Li growth. The inset images are the corresponding enlarged region of the yellow square.
